# 6^th^ *Eurosurveillance* scientific seminar ‘One Health – we are in this together – Viral and bacterial diseases/conditions at the animal–human interface’ at ESCAIDE 2017 in Stockholm

**DOI:** 10.2807/1560-7917.ES.2017.22.41.171019-1

**Published:** 2017-10-19

**Authors:** Eurosurveillance editorial team

**Affiliations:** 1 European Centre for Disease Prevention and Control (ECDC), Stockholm, Sweden

The 6^th^ *Eurosurveillance* scientific seminar will be held as lunchtime seminar on 6 November 2017 during the ESCAIDE conference in Stockholm, Sweden.

The seminar will focus on the interplay of changes in pathogens in animals one the one hand and outbreaks or spread of resistant pathogens in humans on the other. After an introduction by Dr Johanna Takkinen, Head of Disease Programme Food- and Waterborne Diseases and Zoonoses at the European Centre for Disease Prevention and Control, Stockholm, Sweden, Professor Sylvie van der Werf, Head of the Molecular Genetics of RNA Viruses Unit, Institut Pasteur, Paris, France, will talk about viral zoonotic diseases and point out intrinsic and extrinsic factors influencing the dynamics of spillover from animals to humans. Professor Luca Guardabassi, Department of Veterinary Disease Biology, University Copenhagen, Denmark, will then speak about the need for antimicrobial stewardship in animals and highlight the possible role of companion animals in the direct transmission of resistant pathogens to humans resulting from their close physical contact.

The connection between the environment, food, animals and humans with respect to human well-being, sustainable development and health is intuitive. The ‘One Health’ concept encompasses a multidisciplinary, holistic approach to sustain the health and well-being of humans and animals as well as their living environment. One Health has gained increasing attention and importance in the past years owing to the continued growth of the world’s population, predicted to be 9 billion by the year 2050, the opportunities and challenges of a technology-driven, interconnected world, as well as man-made and other changes in our environment that influence the emergence and re-emergence of communicable diseases and conditions.

The seminar will be open to all ESCAIDE participants. Due to the limited number of seats in the room, participants are welcome on a first come, first serve basis.

Read more about ESCAIDE here .

Read more about the Seminar here .

